# Enhanced Glutamatergic Currents at Birth in Shank3 KO Mice

**DOI:** 10.1155/2019/2382639

**Published:** 2019-07-03

**Authors:** Morgane Chiesa, Romain Nardou, Natalia Lozovaya, Sanaz Eftekhari, Roman Tyzio, Damien Guimond, Diana C. Ferrari, Yehezkel Ben-Ari

**Affiliations:** ^1^Department of Neurobiology, Mediterranean Institute of Neurobiology (INMED), Aix-Marseille University, INSERM U1249, 13273 Marseille Cedex 09, France; ^2^Neurochlore, Ben-Ari Institute of Neuroarcheology (IBEN), Fundamental Research Department, Bâtiment Beret-Delaage, Parc Scientifique et Technologique de Luminy, 13288 Marseille Cedex 09, France

## Abstract

Autism spectrum disorders (ASD) are neurodevelopmental disorders induced by genetic and environmental factors. In our recent studies, we showed that the GABA developmental shifts during delivery and the second postnatal week are abolished in two rodent models of ASD. Maternal treatment around birth with bumetanide restored the GABA developmental sequence and attenuated the autism pathogenesis in offspring. Clinical trials conducted in parallel confirmed the usefulness of bumetanide treatment to attenuate the symptoms in children with ASD. Collectively, these observations suggest that an alteration of the GABA developmental sequence is a hallmark of ASD. Here, we investigated whether similar alterations occur in the Shank3 mouse model of ASD. We report that in CA3 pyramidal neurons, the driving force and inhibitory action of GABA are not different in naïve and Shank3-mutant age-matched animals at birth and during the second postnatal week. In contrast, the frequency of spontaneous excitatory postsynaptic currents is already enhanced at birth and persists through postnatal day 15. Therefore, in CA3 pyramidal neurons of Shank3-mutant mice, glutamatergic but not GABAergic activity is affected at early developmental stages, hence reflecting the heterogeneity of mechanisms underlying the pathogenesis of ASD.

## 1. Introduction

Autism spectrum disorders (ASD) are a group of complex neurodevelopmental disorders characterized by three main core symptoms: persistent difficulties in social interactions, deficits in nonverbal communicative behaviors, and the existence of restricted and stereotypic behaviors [1]. For the past decades, the prevalence of these disorders has increased worldwide and is now thought to range between 0.01 and 2%, with males being four times more affected than females [2, 3]. For that reason, ASD have been extensively studied over the past years and its pathophysiological mechanisms have started to be elucidated. It is now commonly accepted that ASD result in part from an imbalance between excitation and inhibition in neural systems [4, 5]. In addition, brain growth alterations are observed in patients with ASD throughout life [6–9]. However, little is known about the early postnatal changes that could take place despite ASD being considered a neurodevelopmental disorder. Yet, this information is essential to understand the impact that a perinatal insult can have on brain developmental sequences and early neuronal activities. Indeed, the “Neuroarcheology” concept [10] implies that misplaced or misconnected neurons generated following an *in utero* insult or genetic mutation produce persistent immature patterns of activity that could account for the pathogenesis of numerous developmental disorders. This provides a unique therapeutic opportunity to treat these disorders by selectively blocking with drugs the perturbing immature currents, hence restoring physiological neuronal activities.

Consistently, our team has discovered major alterations of the GABA developmental sequence in two rodent models of autism [11], the Fragile X and *in utero* valproic acid models that, respectively, mimic a genetic mutation and environmental insult linked to ASD [12, 13]. In these models, the transient neuroprotective oxytocin-mediated GABA excitatory to inhibitory shift at birth is abolished and associated with aberrant GABA excitatory actions in juvenile cortical neurons [11, 14]. These alterations of the polarity and action of GABA are thought to play major roles in the pathogenesis of ASD. Indeed, maternal administration of the NKCC1 chloride importer antagonist bumetanide, a drug that reduces the intracellular concentration of chloride ([Cl^−^]_i_), restores the inhibitory action of GABA at birth and later and attenuates ASD symptoms in offspring. This treatment also abolishes the enhanced glutamatergic network activities reported in these two models [11, 15]. Relying on these observations, clinical trials have successfully shown that bumetanide attenuates the severity of the symptoms in children with ASD [16–19]. Hence, we might expect that the alteration of the GABA developmental sequence is a general hallmark of ASD. To test this hypothesis, we evaluated Shank3-mutant mice, a frequently investigated genetic mouse model of ASD.

Diverse factors have been associated with ASD, with hundreds of genetic mutations thought to account for 10 to 25% of the children with ASD [12, 20]. Although rare with regard to the general population, several mutations of the members of the SH3 and multiple ankyrin repeat domains protein (Shank) family have been identified [21–24] and estimated to be present in about 1% of individuals with ASD [25]. In particular, Shank3 mutations which have the highest frequency among them are the most studied and known to be responsible for the 22q13.3 deletion syndrome (also called Phelan-McDermid syndrome) which falls into the category of ASD [26]. Shank3 is a scaffolding protein localized at the postsynaptic density of glutamatergic synapses that modulates dendritic spine morphology and synaptic signaling through glutamate receptors and interactions with the cytoskeleton [27–31]. This protein is constituted of six domains for protein-protein interactions including the PDZ domain that interacts with the SAP90/PSD95-associated protein 1 and the AMPA receptor 1 subunit to regulate dendritic spine formation and synaptic transmission [32]. Shank3 also binds to Homer [33, 34] and Cortactin [35] to promote the polymerization of the cytoskeleton regulation and mediate synaptic transmission and plasticity [36–38]. Studies on Shank3-mutant mice have shown a global developmental delay and autistic-like features [39–41]. Still, the underlying mechanisms of this phenotype are quite variable with increased or decreased glutamatergic currents depending on the type of neurons and brain areas studied [42–45]. These conflicting results might, in part, be explained by the localization of the mutation on the Shank3 gene that induces specific alterations on one of the six domains for protein-protein interactions [46]. Overall, and independently of the Shank3-mutant mouse model studied, these investigations show that an imbalance between excitation and inhibition is observed in Shank3-mutant mice. However, whether the GABA developmental sequence is altered in Shank3-mutant mice remains to be elucidated.

Here, we report that in CA3 pyramidal neurons, glutamatergic activity is already enhanced at birth and persists through development in Shank3-mutant mice. However, and in contrast to previously evaluated models of ASD, the GABA developmental sequence is unaltered. These results indicate that the disruption of the excitation/inhibition balance in the Shank3-mutant mouse model of ASD may be specifically attributed to an enhancement of glutamatergic activity. Hence, the abolition of the GABA developmental sequence cannot be considered a general hallmark of ASD.

## 2. Material and Methods

### 2.1. Animals

All experiments were carried out in accordance with the European Communities Council Directive (2010/63/EU). Shank3-mutant mice with a disruption of the PDZ domain located at exons 13-16 of the chromosome 15 (Shank3B^−/−^, [45]) were housed in a temperature-controlled environment on a 12 h light cycle (7 am–7 pm) with *ad libitum* access to water and food. Experiments were conducted on Shank3^+/+^ wild-type mice (WT) and Shank3^−/−^ knock-out littermates of both sexes.

### 2.2. Electrophysiological Recordings

This study uses the methods of Chiesa et al. and the description of the electrophysiological recordings partly reproduces their wording [47].

#### 2.2.1. Slice Preparation

Brains were rapidly removed and immersed into ice-cooled (3-5°C) choline with the following composition (in mM): 132.5 choline, 2.5 KCl, 1.2 NaH_2_PO_4_, 3 MgCl_2_, 0.7 CaCl_2_, 25 NaHCO_3_, and 8 glucose, pH 7.4 equilibrated with 95% O_2_ and 5% CO_2_. Horizontal hippocampal slices (400 *μ*m thick) were cut using a Leica VT1200S vibratome (Leica Microsystems, Germany) and transferred to an incubation chamber filled with artificial cerebrospinal fluid (ACSF) with the following composition (in mM): 126 NaCl, 3.5 KCl, 2 CaCl_2_, 1.3 MgCl_2_, 1.2 NaH_2_PO_4_, 25 NaHCO_3_, and 11 glucose, pH 7.4 equilibrated with 95% O_2_ and 5% CO_2_ for at least 1 h prior to recording at room temperature (22-25°C). Slices were placed into the recording chamber and submerged with oxygenated ACSF at a rate of 2-3 mL/min at room temperature. For whole-cell recordings, ACSF of the following composition was used (in mM): 125 NaCl, 3.5 KCl, 2 CaCl_2_, 1 MgCl_2_, 1.2 NaH_2_PO_4_, 26 NaHCO_3_, and 10 glucose. For cell-attached and extracellular field recordings, ACSF of the following composition was used (in mM): 126 NaCl, 3.5 KCl, 2 CaCl_2_, 1.3 MgCl_2_, 1.2 NaH_2_PO_4_, 25 NaHCO_3_, and 11 glucose.

#### 2.2.2. Cell-Attached Recordings

Single GABA_A_ receptor channels were recorded in cell-attached configuration in CA3 pyramidal neurons at P0 and P14-P16 using an EPC-10 amplifier (HEKA Elektronik Dr. Schulze GmbH, Germany). Patch pipette solution contained (in mM) 120 NaCl, 5 KCl, 20 TEA-Cl, 5 4-aminopyridine, 0.1 CaCl_2_, 10 MgCl_2_, 10 glucose, and 10 HEPES-NaOH, pH 7.2-7.3 with GABA at 5 *μ*M. GABA_A_ receptor-mediated currents were recorded for 1-2 min at each imposed voltage from -100 to +60 mV with 10 mV increments. Recordings were digitized and analyzed as described previously [14, 48].

#### 2.2.3. Whole-Cell Recordings of Spontaneous Postsynaptic Currents

These electrophysiological recordings use the methods of Fernandez et al. and our description partly reproduces their wording [49]. Whole-cell recordings of spontaneous glutamatergic and GABAergic currents in CA3 pyramidal neurons at P0 and P15 were performed using an EPC-10 amplifier (HEKA Elektronik Dr. Schulze GmbH, Germany) or a MultiClamp 700B amplifier (Molecular Devices, CA, USA) with custom-made software based on IGOR Pro. Data were acquired at 10 kHz and filtered at 2.4 kHz. Patch pipette solution contained (in mM) 130 K-gluconate, 10 Na-gluconate, 7 NaCl, 4 Mg-ATP, 10 HEPES, 4 phosphocreatine, and 0.3 Na-GTP, pH 7.3 equilibrated with KOH. Spontaneous glutamatergic postsynaptic currents (sEPSCs) were recorded at the reversal potential for GABAergic currents and spontaneous inhibitory postsynaptic currents (sIPSCs) were recorded at the reversal potential for glutamatergic currents. Reversal potentials were experimentally detected by changing the holding potential step by step with 1 mV increments starting at -75 mV for GABAergic and +10 mV for glutamatergic currents. The holding potential at which spontaneous GABAergic and glutamatergic currents were zero was taken as a holding potential for recordings of sEPSCs and sIPSCs, respectively. sIPSCs were recorded for 15 min at a holding potential of +10 ± 2 mV while sEPSCs were recorded for 15 min at a holding potential of −75 ± 2 mV. Data were filtered post recording and analyzed using Mini Analysis 6.0 (Synaptosoft Inc., GA, USA), Clampfit 10.4 (Molecular Devices, CA, USA), and OriginPro (OriginLab, MA, USA).

#### 2.2.4. Whole-Cell Recordings of Miniature Postsynaptic Currents

Whole-cell voltage-clamp recordings of miniature postsynaptic currents in CA3 pyramidal neurons at P14-P16 were performed with a MultiClamp 700B amplifier (Molecular Devices, CA, USA). Miniature excitatory postsynaptic currents (mEPSCs) were recorded with micropipettes containing (in mM) 130 K-gluconate, 10 Na-gluconate, 4 NaCl, 10 HEPES, 4 phosphocreatine, 4 Mg-ATP, and 0.3 Na-GTP at a holding potential of -70 mV in the presence of TTX (1 *μ*M; Abcam, UK) and gabazine (5 *μ*M; Tocris Bioscience, UK). Miniature inhibitory postsynaptic currents (mIPSCs) were recorded with micropipettes containing (in mM) 130 KCl, 10 HEPES, 1.1 EGTA, 0.1 CaCl_2_, 5 phosphocreatine, 4 Mg-ATP, and 0.4 Na-GTP at a holding potential of -70 mV in the presence of TTX (1 *μ*M; Abcam, UK) and CNQX (10 *μ*M; Sigma-Aldrich, MO, USA). All following parameters were controlled to be within acceptable and similar range across recordings: membrane capacitance, resting membrane potential, series resistance, holding current, and firing pattern. Miniature IPSCs and EPSCs were analyzed using Mini Analysis 6.0 (Synaptosoft Inc., GA, USA). The threshold for event detection was defined as 3 times the standard deviation of the noise. Criteria for exclusion were a change in series resistance higher than 20% through recording, baseline oscillations, and distorted shape of synaptic events.

#### 2.2.5. Extracellular Field Recordings

Extracellular field recordings were performed in the CA3 pyramidal layer of hippocampal slices of P16 mice with glass pipettes (Harvard Apparatus, MA, USA) containing ACSF. Spike frequency was recorded for 10 min (control period), then isoguvacine (10 *μ*M; Sigma-Aldrich, MO, USA) was applied in the bath solution for 90 s (isoguvacine period). Following isoguvacine application, spike frequency was recorded for an extra 15 min (wash-out period). Signals were recorded with a low-noise multichannel DAM-80A amplifier (WPI, UK; low-pass filter 1 Hz; high-pass filter 3 kHz; gain ×1000) and digitized online with a Digidata 1400A digitizer (Molecular Devices, CA, USA). Recordings were analyzed using Clampfit 10.4 software (Molecular Devices, CA, USA). The spike detection threshold was defined as three times the standard deviation of the noise recorded in the bath solution. Spike frequency was calculated for control, isoguvacine, and wash-out periods. Slices for which wash-out spike frequency did not come back to control levels (±20% of control) were excluded from this study.

### 2.3. KCC2 Immunohistochemistry

WT and Shank3^−/−^ mice at P14-P15 were anesthetized then transcardially perfused with Antigenfix (Diapath, Italy). Brains were harvested then sliced coronally (70 *μ*m thickness) using a Leica VT1000S vibratome (Leica Microsystems, Germany). Selected sections from WT and Shank3^−/−^ hippocampi were processed in parallel to perform immunohistochemistry under identical conditions. Sections were incubated for 1 h at room temperature (22-25°C) with a solution of 5% normal goat serum (NGS; Jackson ImmunoResearch Laboratories, PA, USA) in phosphate-buffered saline (PBS; Life Technologies, CA, USA) with 0.3% Triton X-100 (Sigma-Aldrich, MO, USA). Then, they were incubated overnight at 4°C in a solution containing the anti-KCC2 antibody (1/800; US Biological, MA, USA) diluted in PBS with 1% NGS and 0.1% Triton X-100. After rinsing in PBS, sections were incubated with the fluorescent-labeled secondary antibody Alexa Fluor 555 (1/1000; Life Technologies, CA, USA) for 1 h at room temperature. Sections were finally mounted on slides and imaged with a confocal microscope (Leica TCS SP5 X, Leica Microsystems, Germany) using identical settings (objective lens, objective aperture, laser power, and photomultiplier gain/offset). KCC2 analysis was performed blind using the open-source platform Fiji (version 1.50e; https://fiji.sc/) [50]. All stack images were split and converted into individual tif files for the analysis. We designed a macro for the Fiji platform to automatize image processing. For each image, the macro applied an Auto Threshold on the foreground to produce a binarized mask of the KCC2 signal. Then, an inverted copy of this mask was generated to isolate the background and subtract it from the original image. Finally, background-subtracted images were processed for mean intensity measurement in the defined regions of interest within CA3: stratum oriens, stratum pyramidale, stratum lucidum, and stratum radiatum. Fluorescence intensity was normalized by the mean intensity of the control condition, and the mean ± SEM was calculated for each condition.

### 2.4. Statistics

Datasets were tested for normality using the D'Agostino and Pearson test prior to any statistical analysis. Data for cell-attached recordings, KCC2 immunohistochemistry, sEPSCs at P0, sIPSCs at P15, mEPSCs amplitude and decay time, and mIPSCs decay time were analyzed with the Mann-Whitney test. sEPSCs at P15, mEPSCs frequency and rise time, and mIPSCs frequency, amplitude, and rise time were analyzed with the two-tailed *t*-test. Extracellular field recordings were analyzed with the repeated measures Friedman test with Dunn's multiple comparison post hoc test.

## 3. Results

### 3.1. The Excitatory to Inhibitory GABA Developmental Sequence Is Not Altered in CA3 Pyramidal Neurons of Shank3^−/−^ Mice

We first evaluated if the GABA developmental sequence was altered in CA3 pyramidal neurons of Shank3-mutant mice, as seen previously in the Fragile X and *in utero* valproic acid rodent models of ASD [11].

**Figure 1:**
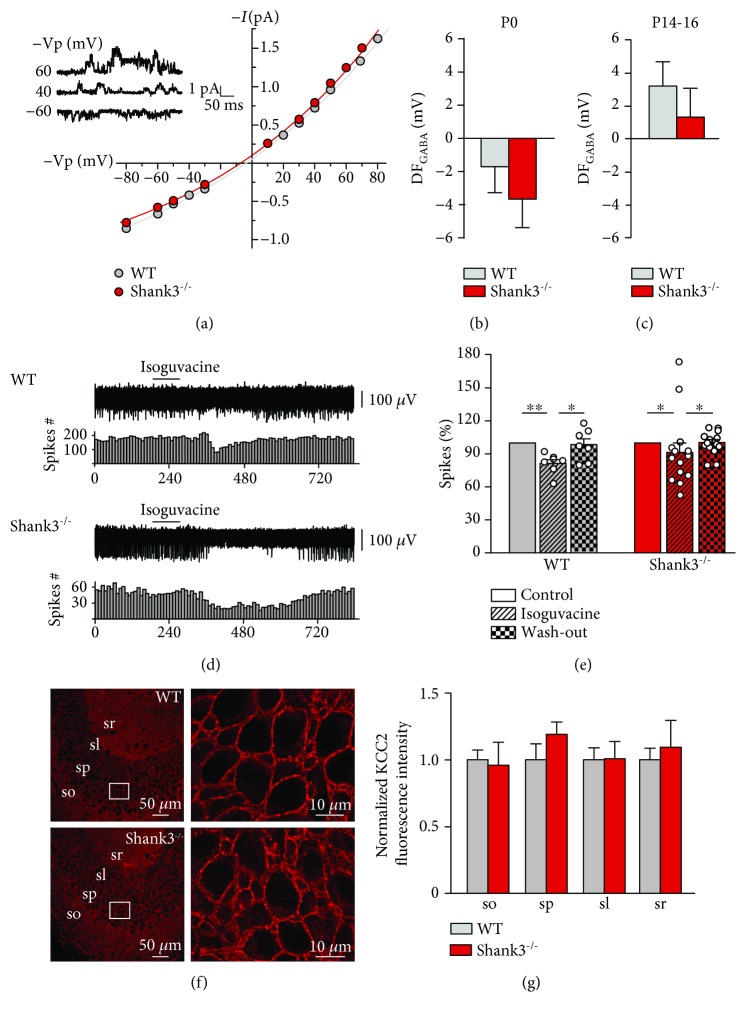
The GABA developmental sequence in CA3 pyramidal neurons is not altered in Shank3^−/−^ mice. (a) Representative *I*/*V* curves from single GABA_A_ receptor channels recordings for WT (in grey) and Shank3^−/−^ (in red) mice at P0. Inset shows representative single GABA_A_ channels recorded in cell-attached configuration at different holding potentials (-Vp) in a P0 WT mouse. (b, c) Average values of DF_GABA_ measured in WT (in grey) and Shank3^−/−^ (in red) mice at birth (b) and at P14-P16 (c). (d) Representative traces of spontaneous extracellular field potentials with corresponding time courses of spike frequency changes after application of isoguvacine (10 *μ*M) for WT and Shank3^−/−^ mice at P16. (e) Histogram of averaged normalized spike frequency in control, isoguvacine, and wash-out periods for WT and Shank3^−/−^ mice at P16. (f) Representative images of KCC2 expression in CA3 hippocampal layers and higher magnification (zoom factor 3) images of KCC2 immunolabeling in the CA3 stratum pyramidale for WT and Shank3^−/−^ mice at P14-P15. (g) Normalized KCC2 fluorescence intensity for WT (in grey) and Shank3^−/−^ (in red) mice in the stratum oriens (so), stratum pyramidale (sp), stratum lucidum (sl), and stratum radiatum (sr) at P14-P15. Data are presented as mean ± SEM, ^∗^*p* < 0.05, ^∗∗^*p* < 0.01. (b) *n* = 32 for WT and *n* = 32 for Shank3^−/−^ mice. (c) *n* = 23 for WT and *n* = 17 for Shank3^−/−^ mice. (e) *n* = 7 for WT and *n* = 14 for Shank3^−/−^ mice. (g) *n* = 5 for WT and *n* = 6 for Shank3^−/−^ mice for all layers except for the stratum radiatum where *n* = 4 for WT.

We performed single GABA_A_ receptor channels recordings to determine if the polarity of GABA was affected in this model. At birth, the driving force of GABA currents (DF_GABA_) was slightly hyperpolarizing for WT (−1.72 ± 1.58 mV) and Shank3^−/−^ mice (−3.65 ± 1.74 mV, *p* = 03411; Figures 1(a) and 1(b) and Supplementary Table 1). Therefore, the transient hyperpolarizing inhibitory activity of GABA at birth is present in Shank3^−/−^ mice. Furthermore, at the end of the second postnatal week, the DF_GABA_ was slightly depolarizing for both WT (3.19 ± 1.47 mV) and Shank3^−/−^ mice (1.31 ± 1.74 mV; *p* = 0.4770; Figure 1(c) and Supplementary Table 1). To address whether this slightly depolarizing DF_GABA_ was associated with an inhibitory or excitatory action of GABA, extracellular field recordings were performed in both WT and Shank3^−/−^ mice. Spike frequency at P16 decreased after the application of isoguvacine, an agonist of GABA_A_ receptors, in WT (81.1 ± 3.46% of control, *p* = 0.0099) and Shank3^−/−^ mice (91.44 ± 8.54% of control, *p* = 0.0421; Figures 1(d) and 1(e)). This effect was transient as spike frequency went back to control levels during the wash-out period (Supplementary Table 2). Therefore, the GABA excitatory to inhibitory shifts are not affected in Shank3^−/−^ mice. Noteworthy, the inhibitory action of GABA persisted after the second postnatal week, as seen with extracellular field recordings performed in mice at P22-P24 (Supplementary Figure 1a). Indeed, the application of isoguvacine decreased spike frequency for WT (74.1 ± 4.40% of control, *p* = 0.0239) and Shank3^−/−^mice (84.22 ± 3.25% of control, *p* = 0.0219; Supplementary Figure 1b and Supplementary Table 3), and spike frequency went back to control levels during the wash-out period.

In addition, since GABA action principally depends on the expression of NKCC1 and KCC2 chloride cotransporters, and the disruption of KCC2 expression is associated with a variety of neurodevelopmental disorders including ASD [11, 51–53], we evaluated its expression in the different layers of CA3 in our model at P14-P15. Similar KCC2 expression levels were found between WT and Shank3^−/−^ mice in the stratum oriens (1 ± 0.07*vs.*0.96 ± 0.17, *p* = 0.6623), stratum pyramidale (1 ± 0.12*vs.*1.19 ± 0.09, *p* = 0.3290), stratum lucidum (1 ± 0.09*vs.*1.01 ± 0.13, *p* = 0.9307), and stratum radiatum (1 ± 0.09*vs.*1.10 ± 0.20, *p* = 0.7619; Figures 1(f) and 1(g) and Supplementary Table 4). These results reinforce the conclusion that the GABA developmental sequence is not disrupted in the Shank3^−/−^ mouse model of ASD.

### 3.2. Spontaneous Glutamatergic Currents Are Already Enhanced at Birth in CA3 Pyramidal Neurons of Shank3^−/−^ Mice

**Figure 2:**
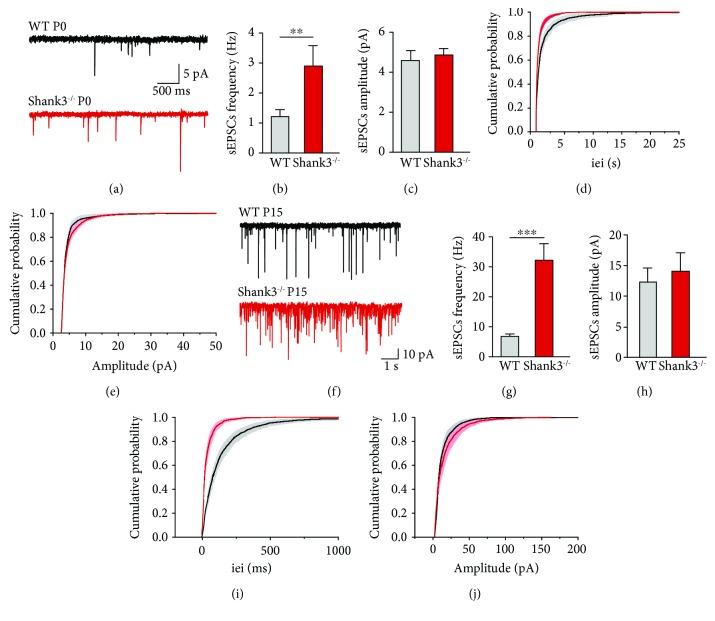
Spontaneous glutamatergic network activity is already enhanced in CA3 pyramidal neurons of Shank3^−/−^ mice at birth. (a) Example of traces of sEPSCs recorded in whole-cell patch clamp at -75 mV in WT (in black) and Shank3^−/−^ (in red) mice at P0. (b, c) Bar graphs show the average frequency (b) and amplitude (c) of sEPSCs at P0. (d, e) Cumulative probability distributions of interevent interval (iei; d) and amplitude (e) in WT and Shank3^−/−^ mice at P0. (f) Representative traces of sEPSCs recorded in whole-cell patch clamp at -75 mV in WT (in black) and Shank3^−/−^ (in red) mice at P15. (g, h) Bar graphs show the average frequency (g) and amplitude (h) of sEPSCs at P15. (i, j) Cumulative probability distributions of iei (i) and amplitude (j) in WT and Shank3^−/−^ mice at P15. Data are presented as mean ± SEM, ^∗∗^*p* < 0.01, ^∗∗∗^*p* < 0.001. (b–e) *n* = 12 for WT and *n* = 13 for Shank3^−/−^ mice. (g–j) *n* = 9 for WT and *n* = 10 for Shank3^−/−^ mice.

Considering the importance of the Shank3 protein for glutamatergic synapses and the increased glutamatergic activity observed in CA3 pyramidal neurons of the Fragile X and *in utero* valproic acid models of ASD [11], we tested if glutamatergic network activity was also altered in Shank3^−/−^ mice by performing whole-cell recordings at P0 (Figure 2(a)) and P15 (Figure 2(f)).

At birth, glutamatergic network activity was enhanced in CA3 pyramidal neurons of Shank3^−/−^ mice, as seen by an increased frequency of spontaneous excitatory postsynaptic currents (sEPSCs; 2.90 ± 0.68 Hz*vs.*1.21 ± 0.24 Hz, *p* = 0.008; Figures 2(b) and 2(d) and Supplementary Table 5) even though the amplitude was similar for WT and Shank3^−/−^ mice (4.60 ± 0.49 pA*vs.*4.86 ± 0.32 pA, respectively, *p* = 0.4288; Figures 2(c) and 2(e) and Supplementary Table 5). Interestingly, this increased glutamatergic network activity persisted through development, as it is still present during the second postnatal week. Indeed, in CA3 pyramidal neurons of Shank3^−/−^ mice at P15, the frequency of sEPSCs was increased compared to WT mice (32.19 ± 5.52 Hz*vs.*6.80 ± 0.81 Hz, respectively, *p* = 0.0005; Figures 2(g) and 2(i) and Supplementary Table 5) while the amplitude was similar (14.35 ± 3.10 pA*vs.*12.56 ± 2.35 pA, *p* = 0.6558; Figures 2(h) and 2(j) and Supplementary Table 5). In addition, the frequency, amplitude, and decay time of miniature excitatory postsynaptic currents (mEPSCs) were not different for P14-P16 Shank3^−/−^ mice compared to WT, even if a slight change in the rise time was observed (Supplementary Figure 2 and Supplementary Table 6). These results suggest that the alteration of the glutamatergic spontaneous activity is action potential driven.

Therefore, as observed in the Fragile X and *in utero* valproic rodent models of ASD, glutamatergic network activity is already altered at birth and persists at P15 in Shank3^−/−^ mice.

### 3.3. Spontaneous GABAergic Currents in CA3 Pyramidal Neurons Are Not Altered in Shank3^−/−^ Mice during Development

**Figure 3:**
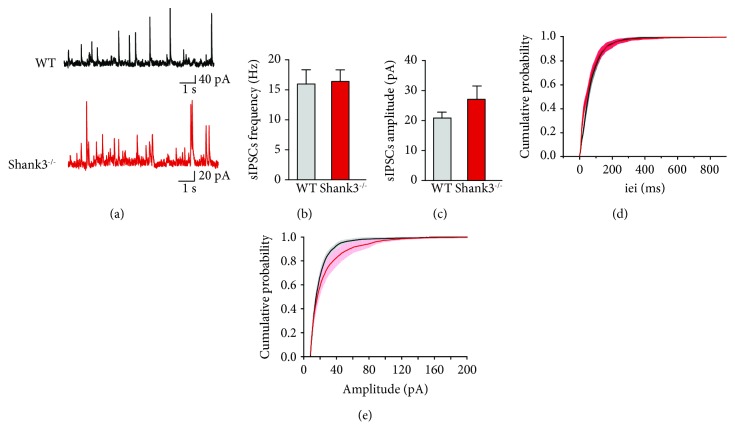
Spontaneous GABAergic network activity is not altered in CA3 pyramidal neurons of Shank3^−/−^ mice at P15. (a) Example of traces of sIPSCs recorded in whole-cell patch clamp at +10 mV in WT (in black) and Shank3^−/−^ (in red) mice at P15. (b, c) Bar graphs show the average frequency (b) and amplitude (c) of sIPSCs. (d, e) Cumulative probability distributions of iei (d) and amplitude (e) in WT and Shank3^−/−^ mice. Data are presented as mean ± SEM. (b–e) *n* = 9 for WT and *n* = 7 for Shank3^−/−^ mice.

Because it is commonly accepted that ASD result from an imbalance between excitation and inhibition, we further assessed if spontaneous GABAergic network activity was also affected in Shank3^−/−^ mice using whole-cell patch clamp at P15 (Figure 3(a)). The frequency of spontaneous inhibitory postsynaptic currents (sIPSCs) in CA3 pyramidal neurons was similar for Shank3^−/−^ and WT mice (16.4 ± 1.94 Hz*vs.*15.99 ± 2.37 Hz, respectively, *p* = 0.7577; Figures 3(b) and 3(d) and Supplementary Table 7). Moreover, the amplitude of sIPSCs did not differ between Shank3^−/−^ and WT mice (27.11 ± 4.46 pA*vs.*20.89 ± 1.95 pA, respectively, *p* = 0.1416; Figures 3(c) and 3(e) and Supplementary Table 7). Also, the frequency and amplitude of miniature inhibitory postsynaptic currents (mIPSCs) were not altered in P14-P16 Shank3^−/−^ mice compared to WT, even if the rise time and decay time were slightly affected, suggesting that a change in GABA_A_ receptor subunits might exist in CA3 pyramidal cells of Shank3^−/−^ mice at P14-P16 (Supplementary Figure 3 and Supplementary Table 8).

Thus, GABA network activity is not altered in CA3 pyramidal neurons of Shank3^−/−^ mice, suggesting that the disruption of the excitation/inhibition balance in this model of ASD may generally be attributed to an enhancement of the glutamatergic activity.

## 4. Discussion

An essential issue to understand and treat ASD and related disorders is to determine whether developmental sequences are already deviated during early postnatal life and how they lead to aberrant activity generated by neuronal assemblies. Indeed, we have previously suggested that these deviations, rather than the genetic mutations and/or environmental insults *per se*, are responsible for the deleterious physiological and behavioral sequels observed [11]. This conceptual notion has already been supported by the findings in several models of genetic mutations inducing migration disorders, where misplaced neurons have been found to have immature features that are absent in age-matched naïve neurons [54–56].

Among brain developmental sequences, the shift from an excitatory to inhibitory action of GABA is probably one of the best investigated ones and its alteration has been shown to lead to important consequences in preclinical and clinical paradigms. In physiological conditions, while the transient GABA shift at birth is oxytocin-mediated, the shift that takes place during the second postnatal week is under the control of the developmental expression of two main chloride cotransporters: NKCC1 and KCC2. The increase in KCC2 expression during the second postnatal week results in a decreased [Cl^−^]_i_ and a slightly depolarizing GABA activity that is associated with an inhibitory action [57]. Interestingly, CA3 pyramidal neurons recorded in two animal models of ASD were found to have a pathogenic depolarizing activity combined with an excitatory action of GABA shortly after birth and through development [11, 52]. Here, our aim was to determine whether this deviation of the GABA developmental sequence could be considered a general hallmark of ASD. To do so, we studied the well-investigated hippocampal network, and specifically CA3 pyramidal neurons, in a widely used genetic model of ASD, the Shank3^−/−^ mouse model.

Even if Shank3^−/−^ mice have been reported to perform at similar levels as controls for the Morris water maze, a task that is widely used to evaluate hippocampal-dependent learning and memory [45], a recent study using this same mouse model reported deficits in reference memory in the 4/8 radial arm water maze that may underlie learning disabilities [58]. In addition, an impossibility to complete pretraining in an operant learning test, possibly due to high self-grooming and low locomotor activity, suggests challenges in assessing complex tasks in Shank3^−/−^ mice [59]. Furthermore, even though the hippocampus is primarily known for its modulation of spatial navigation and episodic memory, studies have also shown that this structure processes information relative to affective and motivated behaviors [60, 61]. Interestingly, amygdala inputs projecting towards the ventral hippocampus can modulate social behaviors [62], while the activation of the relaxin-3 receptor in the ventral hippocampus results in increased anxiety behaviors and social avoidance [63]. Thus, alterations in anxiety and social interaction behaviors reported in studies using Shank3^−/−^ mice [45, 64, 65] might be due in part to an impairment in hippocampal activity, and evaluating developmental alterations during the first weeks of life in this structure could shed some light into the underlying mechanisms of this phenotype.

Our results did not confirm the hypothesis that deviations to the GABA developmental shifts are a hallmark of ASD since the GABA developmental sequence in CA3 pyramidal neurons was not altered in the Shank3 mouse model of ASD. This finding is still of particular importance since there is a general agreement that in ASD and related developmental disorders, an imbalance between excitation and inhibition is present in diverse brain regions [4, 66]. Yet, the exact nature of the mechanisms underlying this imbalance is not well understood nor seems to be homogenous. Indeed, several studies using Shank3-mutant mice showed little or no alterations of GABAergic currents [39, 45, 67]. However, in the BTBR T+^tf^/J mouse model of idiopathic ASD and Shank3B^−/−^ mutated mice, several markers of GABAergic signals including the number of parvalbumin puncta surrounding NeuN-positive pyramidal cell bodies and the intensity of the parvalbumin puncta are reduced in the insular cortex [68]. In addition, the number of parvalbumin-positive cells and parvalbumin mRNA and protein levels are also decreased in the striatum of Shank3^−/−^ mice [69], suggesting that deficits in the parvalbumin circuitry might be a signature for ASD. Still, parvalbumin defects seem restricted to specific brain regions since the number of parvalbumin positive cells and parvalbumin protein levels do not appear modified in the cortex and hippocampus of Shank3^−/−^ mice [69]. This disparity of results could be attributed to the heterogeneous expression of Shank3 isoforms in the brain that may explain the differences reported based on the Shank3 rodent model of ASD and the structure studied [46]. This also reflects the heterogeneity of alterations associated with ASD (and ASD models) and its triggering factors. Importantly, developmental alterations have not been systematically investigated, adding a significant caveat to the conclusions that can be drawn specifically when studying developmental disorders. Hence, our results contribute the important observation that right after birth and during early postnatal life, the polarity of GABA in CA3 pyramidal neurons appears not to be affected by the Shank3 mutation. This result suggests that GABAergic signals that are instrumental for the orchestration and maturation of developmental networks and behavioral relevant signals might not be affected in Shank3-mutant mice. Nevertheless, we observed a slight alteration of the kinetic of miniature inhibitory postsynaptic currents in Shank3-mutant mice at the end of the second postnatal week. During development, a switch in the expression of GABA_A_ receptor subunits has been reported in the brain of rodents, with an upregulation of the expression of *α*2 and *α*5 subunits between P10 and P30 in the hippocampus [70]. Interestingly, decreasing the expression of GABA_A_*α*5 subunit in a mouse model of Down syndrome attenuates the cognitive, electrophysiological, and morphological defects observed [71]. Still, in Cntnap2^−/−^, Shank3^−/−^, and 16p11.2^−/−^ mice models of ASD [72], GABA_A_ receptor density was not affected in adults. Moreover, the frequency of mIPSCs is increased in CA1 pyramidal cells of P23-P27 Shank3^−/−^ mice, while this frequency is decreased in layer 2/3 cells of the medial prefrontal cortex of these P39-P54 mice [44]. These results suggest that GABA_A_ receptor subunit expression and synaptic transmission are differently affected depending on the age, structure, type of mutation, or ASD model studied.

Our results show that glutamatergic postsynaptic currents are already enhanced in CA3 pyramidal neurons of Shank3^−/−^ mice at birth. Previous studies have shown more consistently alterations of glutamatergic AMPA and/or NMDA receptor-mediated signaling [73–75]. This is in keeping with the role and localization of the Shank3 protein. Indeed, Shank3, a scaffolding protein located at the postsynaptic density of glutamatergic synapses, is required for proper protein-protein interactions involved in cytoskeleton regulation, dendritic spine formation, synaptic transmission, and plasticity [46]. Here, we demonstrated that, in addition to the alterations found in other brain structures [41, 45, 73, 75], the imbalance associated to ASD is due to an alteration of glutamatergic rather than GABAergic signals that is already present at birth in CA3 pyramidal neurons. Determining if this pattern of activity is also present in other brain regions would be interesting so as to evaluate the universal nature of this phenotype in Shank3^−/−^ mice. Further investigations will be needed at earlier stages to determine whether glutamatergic activity is already enhanced *in utero* or if the effect of the Shank3 mutation is worsened by birth in order to target a critical window for future therapeutic treatments aiming to restore these deviations.

## 5. Conclusion

A better understanding of ASD is crucial to identify hallmarks of the disease that will allow an earlier detection and improve therapeutic treatments available for this pathology. In our previous work in two rodent models of ASD, we found that the disruption of the GABA developmental sequence was associated with an enhancement of glutamatergic network activity in CA3 pyramidal neurons. Interestingly, maternal pretreatment with bumetanide restored the GABA developmental sequence and led to the abolition of enhanced glutamatergic network activity and attenuation of autistic-like behaviors in offspring, suggesting that this component is essential in the pathogenesis and might be a common feature in ASD. Even though the glutamatergic network activity of CA3 pyramidal neurons was already enhanced at birth in the Shank3^−/−^ mouse model of ASD, the GABA developmental sequence and GABA network activity were not disrupted, averting the hypothesis that bumetanide will work as a potential treatment.

## Data Availability

Additional data used to support the findings of this study are accessible in the supplementary materials.

## Supplementary Materials

Supplementary Materials The supplementary file submitted is a 6-page document that contains tables and figures. It is presented as a final version ready to be published along with the article.
