# Supplementary material for: Enhanced Glutamatergic Currents at Birth in Shank3 KO Mice
Source: Neural Plast. 2019 Jul 3;2019:2382639. doi: 10.1155/2019/2382639 (PMC6636579; doi:10.1155/2019/2382639)

Supplementary Material

Enhanced Glutamatergic Currents at Birth in Shank3 KO Mice

Morgane Chiesa[1,2†], Romain Nardou[2†], Natalia Lozovaya[2], Sanaz Eftekhari[1,2], Roman Tyzio[1,2], Damien Guimond[2], Diana C. Ferrari[2], Yehezkel Ben-Ari[1,2*]

[1]Department of Neurobiology, Mediterranean Institute of Neurobiology (INMED), Aix-Marseille University, INSERM U1249, 13273 Marseille cedex 09, France.

[2]Neurochlore, Ben-Ari Institute of Neuroarcheology (IBEN), Fundamental Research Department, bâtiment Beret-Delaage, Parc scientifique et technologique de Luminy, 13288 Marseille cedex 09, France.

[†]These authors contributed equally to the work.
*Correspondence should be addressed to Yehezkel Ben-Ari; yehezkel.ben-ari@inserm.fr

Supplementary Tables

Supplementary Table 1. GABA polarity during development in CA3 pyramidal neurons is similar for WT and $Shank3^{-/-}$ mice. Numbers correspond to Figure 1b and 1c. Statistics are presented by the Mann-Whitney test.

		WT	$Shank3^{-/-}$	Statistics
DF_{GABA} (mV) at P0	n<br>mean ± SEM	32<br>-1.72 ± 1.58	32<br>-3.65 ± 1.74	p=0.3411
DF_{GABA} (mV) at P14-P16	n<br>mean ± SEM	23<br>3.19 ± 1.47	17<br>1.31 ± 1.74	p=0.4770

Supplementary Table 2. Application of the $GABA_A$ receptor agonist isoguvacine (10 µM) decreases the frequency of spikes (% of control) in extracellular field recordings of CA3 hippocampal slices at P16. Numbers correspond to Figure 1e. Statistics are presented by the repeated measures Friedman test with Dunn's multiple comparisons post hoc test.

	Isoguvacine (%), mean ± SEM	Wash-out (%), mean ± SEM	Isoguvacine *vs.* control	Isoguvacine *vs.* wash-out	Wash-out *vs.* control
WT (n=7)	81.1 ± 3.46	98.42 ± 5.32	**p=0.0099**	**p=0.0226**	p>0.9999
$Shank3^{-/-}$ (n=14)	91.44 ± 8.54	100.4 ± 2.69	**p=0.0421**	**p=0.0245**	p>0.9999

Supplementary Table 3. Application of the $GABA_A$ receptor agonist isoguvacine (10 µM) decreases the frequency of spikes (% of control) in extracellular field recordings of CA3 hippocampal slices at P22-P24. Numbers correspond to Supplementary Figure 1b. Statistics are presented by the repeated measures Friedman test with Dunn's multiple comparisons post hoc test.

	Isoguvacine (%), mean ± SEM	Wash-out (%), mean ± SEM	Isoguvacine *vs.* control	Isoguvacine *vs.* wash-out	Wash-out *vs.* control
WT (n=12)	74.1 ± 4.40	111.1 ± 2.70	**p=0.0239**	**p<0.0001**	p=0.1237
$Shank3^{-/-}$ (n=10)	84.22 ± 3.25	106.9 ± 2.84	**p=0.0219**	**p=0.0002**	p=0.5391

Supplementary Table 4. KCC2 expression is not altered in the CA3 hippocampal layers of P14-P15 $Shank3^{-/-}$ mice. Numbers correspond to Figure 1g. Statistics are presented by the Mann-Whitney test.

			WT	$Shank3^{-/-}$	Statistics
Normalized KCC2 fluorescence intensity	Stratum oriens	n<br>mean ± SEM	5<br>1 ± 0.07	6<br>0.96 ± 0.17	p=0.6623
	Stratum pyramidale	n<br>mean ± SEM	5<br>1 ± 0.12	6<br>1.19 ± 0.09	p=0.3290
	Stratum lucidum	n<br>mean ± SEM	5<br>1 ± 0.09	6<br>1.01 ± 0.13	p=0.9307
	Stratum radiatum	n<br>mean ± SEM	4<br>1 ± 0.09	6<br>1.10 ± 0.20	p=0.7619

Supplementary Table 5. Glutamatergic network activity is already enhanced at birth in CA3 pyramidal neurons of $Shank3^{-/-}$ mice. Numbers correspond to Figure 2b, 2c, 2g and 2h. Statistics are presented by the Mann-Whitney test for sEPSCs at P0 and by the two-tailed t-test for sEPSCs at P15.

		WT	$Shank3^{-/-}$	Statistics
Figures 2b and 2c - P0				
sEPSCs frequency (Hz)	n<br>mean ± SEM	12<br>1.21 ± 0.24	13<br>2.90 ± 0.68	**p=0.0080**
sEPSCs amplitude (pA)	n<br>mean ± SEM	12<br>4.60 ± 0.49	13<br>4.86 ± 0.32	p=0.4288
Figures 2g and 2h - P15				
sEPSCs frequency (Hz)	n<br>mean ± SEM	9<br>6.80 ± 0.81	10<br>32.19 ± 5.52	**p=0.0005**
sEPSCs amplitude (pA)	n<br>mean ± SEM	9<br>12.56 ± 2.35	10<br>14.35 ± 3.10	p=0.6558

Supplementary Table 6. The frequency and amplitude of miniature excitatory postsynaptic currents (mEPSCs) are not altered, but their kinetic is slightly affected in CA3 pyramidal neurons of Shank3$^{-/-}$ mice at P14-P16. Numbers correspond to Supplementary Figure 2b, 2c, 2e and 2f. Statistics are presented by the two-tailed t-test for frequency and rise time, and by the Mann-Whitney test for amplitude and decay time.

Supplementary Figure 2	WT (n=14), mean ± SEM	Shank3$^{-/-}$ (n=12), mean ± SEM	Statistics
Frequency (Hz)	2.13 ± 0.15	2.55 ± 0.26	p=0.1572
Amplitude (pA)	9.16 ± 0.63	9.31 ± 0.50	p=0.5952
Rise time (ms)	2.10 ± 0.10	2.54 ± 0.17	**p=0.0303**
Decay time (ms)	8.47 ± 0.63	9.74 ± 0.71	p=0.3952

Supplementary Table 7. GABAergic network activity is not altered in CA3 pyramidal neurons of Shank3$^{-/-}$ mice at P15. Numbers correspond to Figure 3b and c. Statistics are presented by the Mann-Whitney test.

	WT (n=9), mean ± SEM	Shank3$^{-/-}$ (n=7), mean ± SEM	Statistics
sIPSCs frequency (Hz)	15.99 ± 2.37	16.4 ± 1.94	p=0.7577
sIPSCs amplitude (pA)	20.89 ± 1.95	27.11 ± 4.46	p=0.1416

Supplementary Table 8. The frequency and amplitude of miniature inhibitory postsynaptic currents (mIPSCs) are not altered, but the kinetics are slightly affected in CA3 pyramidal neurons of Shank3$^{-/-}$ mice at P14-P16. Numbers correspond to Supplementary Figure 3b, 3c, 3e and 3f. Statistics are presented by the two-tailed t-test for frequency, amplitude and rise time, and by the Mann-Whitney for the decay time.

Supplementary Figure 3	WT (n=13), mean ± SEM	Shank3$^{-/-}$ (n=15), mean ± SEM	Statistics
Frequency (Hz)	6.36 ± 0.81	5.93 ± 0.57	p=0.6620
Amplitude (pA)	27.75 ± 1.34	25.97 ± 1.17	p=0.3222
Rise time (ms)	1.59 ± 0.06	1.41 ± 0.04	**p=0.0151**
Decay time (ms)	16.78 ± 0.46	15.11 ± 0.53	**p=0.0376**

Supplementary Figures

Supplementary Figure 1. The inhibitory action of GABA is persistent in CA3 pyramidal neurons of WT and Shank3$^{-/-}$ mice. (a) Representative traces of spontaneous extracellular field potentials with corresponding time courses of spike frequency changes after application of isoguvacine (10 µM) in a WT and Shank3$^{-/-}$ mouse at P22. (b) Histogram of averaged normalized spike frequency in control, isoguvacine and wash-out periods for WT and Shank3$^{-/-}$ mice at P22-P24. Data are presented as mean ± SEM, *p<0.05, ***p<0.001, ****p<0.0001 with n=12 for WT and n=10 for Shank3$^{-/-}$ mice.

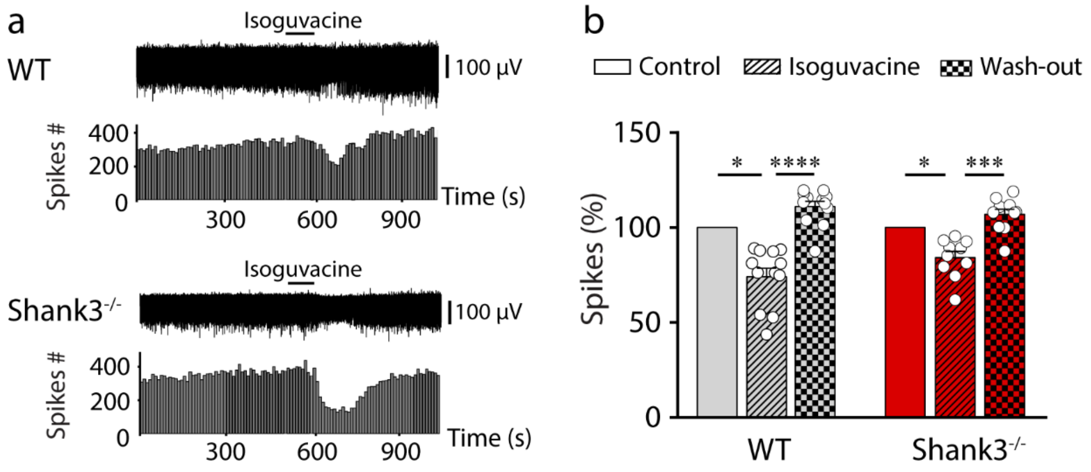

Supplementary Figure 2. Miniature excitatory synaptic activity is slightly affected in Shank3$^{-/-}$ mice at P14-P16. (a) Representative traces of miniature excitatory postsynaptic currents (mEPSCs) in WT (in black) and Shank3$^{-/-}$ (in red) mice recorded at -70 mV in CA3 pyramidal neurons. (b-c) Bar graphs show the averaged frequency (b) and amplitude (c) of mEPSCs in WT and Shank3$^{-/-}$ conditions. (d) Average traces of mEPSCs recorded from all CA3 pyramidal neurons in WT and Shank3$^{-/-}$ mice. The amplitude of the two average traces shown was scaled from baseline to peak. For each individual cell, mEPSCs were aligned on the start of the rising phase to compute the cell's average trace, which was used to measure the mEPSCs rise and decay times. (e-f) Bar graphs show the averaged rise time (e) and decay time (f) of mEPSCs in WT and Shank3$^{-/-}$ conditions. Data are presented as mean ± SEM, *p<0.05 with n=14 for WT and n=12 for Shank3$^{-/-}$ mice.

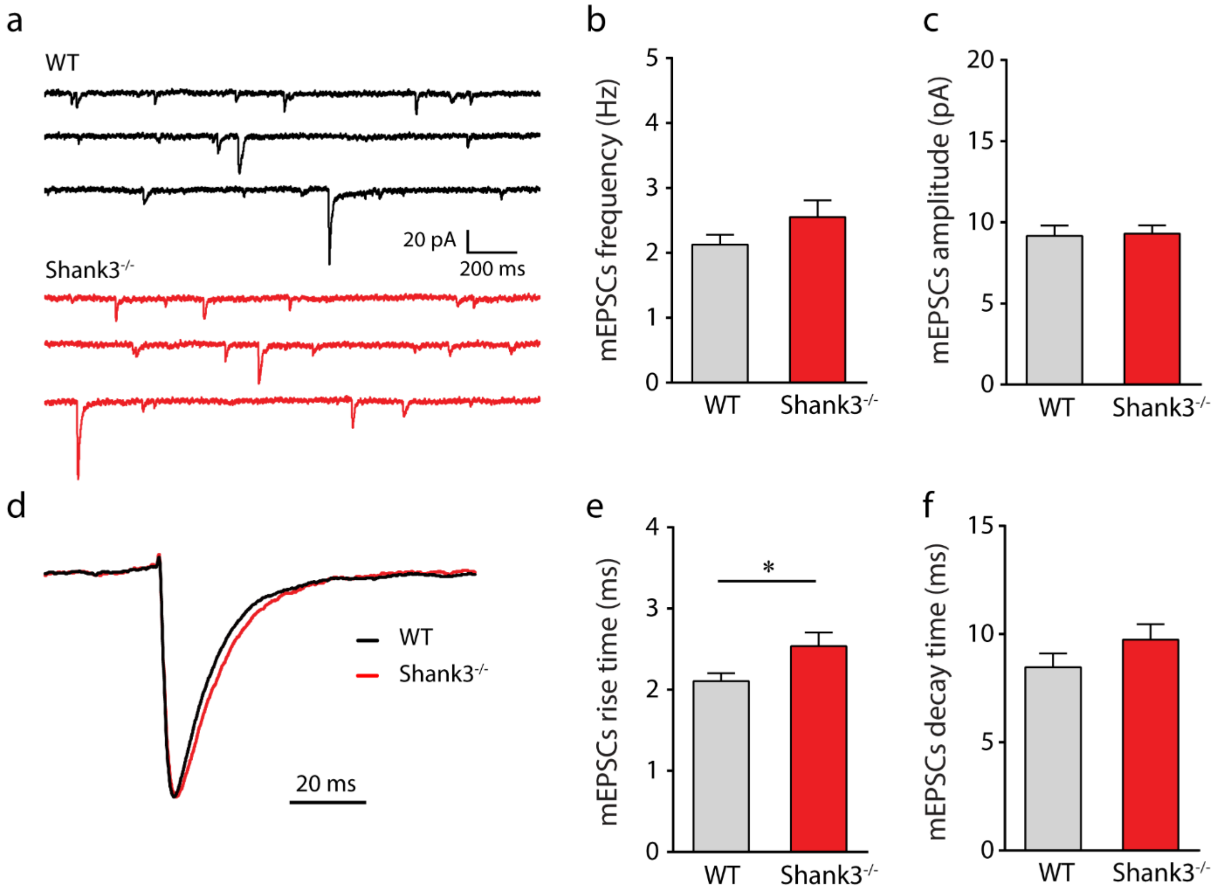

Supplementary Figure 3. Miniature inhibitory synaptic activity is slightly affected in Shank3$^{-/-}$ mice at P14-P16. (a) Representative traces of miniature inhibitory postsynaptic currents (mIPSCs) in WT (in black) and Shank3$^{-/-}$ (in red) mice recorded at -70 mV in CA3 pyramidal neurons. (b-c) Bar graphs show the averaged frequency (b) and amplitude (c) of mIPSCs in WT and Shank3$^{-/-}$ conditions. (d) Average traces of mIPSCs recorded from all CA3 pyramidal neurons in WT and Shank3$^{-/-}$ mice. The amplitude of the two average traces was scaled from baseline to peak. For each individual cell, mIPSCs were aligned on the start of the rising phase to compute average traces which were used to measure mIPSCs rise and decay times. (e-f) Bar graphs show the averaged rise time (e) and decay time (f) of mIPSCs in WT and Shank3$^{-/-}$ conditions. Data are presented as mean ± SEM, *p<0.05 with n=13 for WT and n=15 for Shank3$^{-/-}$ mice.

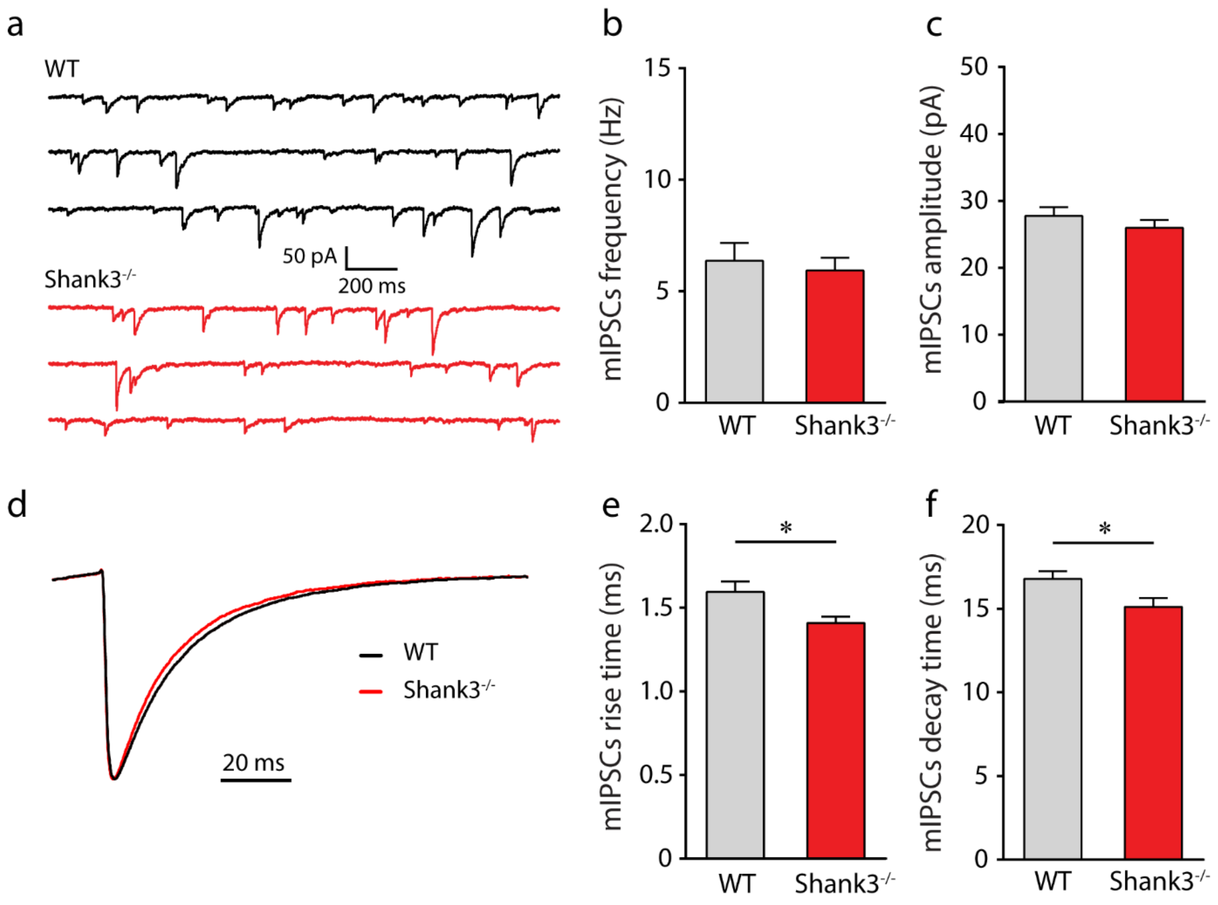

Supplement: Supplementary Materials — The supplementary file submitted is a 6-page document that contains tables and figures. It is presented as a final version ready to be published along with the article. [file 2382639.f1.pdf]
